# Impact of diagnostic genetic testing for familial dementia: experiences of patients and relatives

**DOI:** 10.1186/s13195-026-02000-z

**Published:** 2026-03-17

**Authors:** Jetske van der Schaar, Leonie N. C. Visser, Eva C. A. Asscher, Yolande A. L. Pijnenburg, Christa M. de Geus, Wiesje M. van der Flier, Annelien L. Bredenoord, Mariette A. van den Hoven, Ellen M. A. Smets, Sven J. van der Lee

**Affiliations:** 1 Genomics of Neurodegenerative Diseases and Aging, Department of Human Genetics, Amsterdam UMC location VUmc, Amsterdam, The Netherlands; 2 Alzheimer Center Amsterdam, Neurology, Vrije Universiteit Amsterdam, Amsterdam UMC location VUmc, Amsterdam, The Netherlands; 3 Neurodegeneration, Amsterdam Neuroscience, Amsterdam, The Netherlands; 4 Department of Medical Psychology, Amsterdam UMC location AMC, Amsterdam, The Netherlands; 5 Quality of Care, Amsterdam Public Health, Amsterdam, The Netherlands; 6 Department of Bioethics and Health Humanities, Julius Center for Health Sciences and Primary Care, University Medical Center Utrecht, Utrecht University, Utrecht, The Netherlands; 7 Department of Ethics, Law and Humanities, Amsterdam UMC, Amsterdam, The Netherlands; 8 Clinical Genetics, Department of Human Genetics, Amsterdam UMC location VUmc, Amsterdam, The Netherlands; 9 Alzheimer Nederland, Amersfoort, The Netherlands; 10 Department of Epidemiology & Data sciences, Vrije Universiteit Amsterdam, Amsterdam UMC location VUmc, Amsterdam, The Netherlands; 11 Erasmus School of Philosophy, Erasmus University Rotterdam, Rotterdam, The Netherlands

**Keywords:** Monogenic causes, Pathogenic mutations, Dna-diagnostics, Genetic testing, Symptomatic testing, Familial dementia, Alzheimer’s disease, Frontotemporal dementia, Vascular dementia, Lewy body dementia, Patient experience, Understanding, Psychosocial impact, Behavioral impact, Regret

## Abstract

**Background:**

While memory clinic patients express interest in diagnostic genetic testing for monogenic causes of dementia, its personal consequences remain unclear. We explored the psychosocial and behavioral impact on patients and relatives.

**Methods:**

In this mixed-methods study, we examined 31 patients meeting eligibility criteria for genetic testing as part of their diagnostic work-up at Alzheimer Center Amsterdam. Patients were 45% female, and aged 61 ± 8 (MMSE = 23 ± 5, 25 dementia [15 AD, 4 PPA, 6 other], 1 MCI, 1 SCD, 4 other/undetermined). Twenty-five tested negative, and six positive. Per case, either the patient, one or more relatives, or both were included (3/31 patient only; 5/31 relative(s) only; 23/31 both), yielding 26 patients and 29 relatives (55% female, and aged 54 ± 12). Participants completed questionnaires assessing psychosocial and behavioral factors at first visit, one week post-counseling, and one week and three months post-disclosure. We used linear mixed models to calculate effects of group, time, and interaction, with patients and relatives analyzed separately. In addition, 8 patients and 10 relatives participated in 13 semi-structured interviews, analyzed inductively.

**Results:**

Average anxiety, depression and distress levels remained below clinical threshold in patients and relatives. Anxiety was lower in patients one week and three months post-disclosure, and lower in relatives one week post-disclosure. At baseline, positive patients reported lower social support, and over time they discussed genetic testing less often with others. Positive patients more often reported having registered for research participation. Patients’ decision regret was low and independent of group. Interviews revealed that participants receiving negative results felt relieved their offspring were not at genetic risk, but some remained uncertain about the cause of their disease. Those receiving positive results experienced emotional distress about potential implications for their children, yet appreciated having clarity. Participants related to positive cases described challenges in receiving emotional support.

**Conclusions:**

While diagnostic genetic testing was well tolerated and valued, it also revealed complex emotional, relational, and practical consequences for patients and families, indicating a need for psychosocial support. As this was an exploratory study, findings should be interpreted cautiously and validated in future research.

**Supplementary Information:**

The online version contains supplementary material available at 10.1186/s13195-026-02000-z.

## Background

Dementia is a complex and heterogeneous clinical syndrome, characterized by progressive cognitive decline that interferes with daily functioning. It can result from a wide range of underlying pathologies, leading to Alzheimer’s disease (AD), Vascular dementia (VaD), dementia with Lewy bodies (DLB), and Frontotemporal dementia (FTD). The majority of cases occur later in life and are highly polygenic, involving many common genetic variants, each contributing a small effect to overall disease susceptibility [1]. However, a minority of patients present with early-onset forms, originating from a single mutation in a specific gene, e.g., *PSEN1*,* PSEN2* and *APP* (AD), *MAPT*,* GRN*, and *C9orf72* (FTD) or *NOTCH3*,* HTRA1* and COL4A1/2 (VaD) [2–5]. These monogenic causes are typically inherited in an autosomal dominant manner and exhibit high to full penetrance, implying children have a 50% risk of inheriting the pathogenic allele and subsequently developing symptoms at a relatively young age [6]. As associated pedigrees often show successive generations affected, these conditions are commonly referred to as familial dementia.

Testing for these genetic causes offers clinicians a valuable tool for establishing a definitive molecular diagnosis, particularly in cases of early-onset or atypical dementia where the underlying etiology is unclear [7]. This can avoid protracted delays, facilitate acceptance within families, and allow time to organize care and support [8]. Yet despite the increasing feasibility and affordability of screening for nearly all established monogenic causes [9] in well-resourced healthcare settings, its use in clinical practice remains limited, with implementation varying considerably, both between and within countries [10, 11]. Broader uptake is tempered by uncertainty about how diagnostic testing affects people with dementia and their families beyond its biomedical value [10, 11]. Cognitive impairment may limit some patients’ capacity for informed consent and comprehension of genetic findings, complicating communication of this information to relatives [10, 11]. A positive result may have far-reaching consequences for those at risk, enabling informed decisions about reproductive choices, research participation, and future planning, while potentially affecting emotional well-being, straining relationships, and eliciting stigma, discrimination or suicidality [12, 13]. Conversely, not identifying a pathogenic variant may offer some reassurance but can also leave important questions unanswered [14]. Despite growing attention to the ethical, legal, and social implications of genetic testing in neurodegenerative diseases, empirical data on the real-world experiences of patients undergoing testing in clinical memory services are limited. Most research to date has focused on predictive testing in at-risk family members [12], rather than symptomatic patients undergoing testing as part of their diagnostic trajectory.

To support the responsible use of genetic testing in memory clinics, we previously developed data-driven criteria for identifying patients eligible for testing for monogenic causes of dementia [15]. These are intended to guide clinical decision-making by selecting individuals with a high likelihood of carrying a pathogenic variant. In subsequent work, we examined interest in and attitudes toward diagnostic testing among patients and their families. About half of those who were offered this opportunity chose to pursue it, with decisions mainly shaped by personal values, perceived utility, and concerns about potential psychosocial impacts on relatives [16].

Building on this work, we conducted a mixed-methods study to explore the personal impact of diagnostic genetic testing for monogenic causes of dementia on memory clinic patients and their relatives. We aimed to examine psychosocial outcomes, including comprehension of test result, psychological outcomes, communication and support, decision regret and satisfaction with care, as well as behavioral changes operationalized as personal actionability. By focusing on symptomatic individuals in a diagnostic setting, this study offers novel insight into the real-world implications of genetic testing in memory clinics and informs development of patient-centered care.

## Methods

### Study design

In this observational study, we employed a mixed-methods approach to explore self-reported outcomes and experiences of patients and their family members regarding genetic testing for monogenic causes of dementia. The protocol was approved by the Ethics Committee of Amsterdam UMC (#2021.0534) on October 1st, 2021, and all methods were carried out in accordance with relevant guidelines and regulations. This research was conducted at a tertiary memory clinic, serving relatively young patients with complex clinical presentations, with the vast majority (> 95%) providing informed consent for the use of their clinical data for research purposes [17, 18]. 

**Fig. 1:**
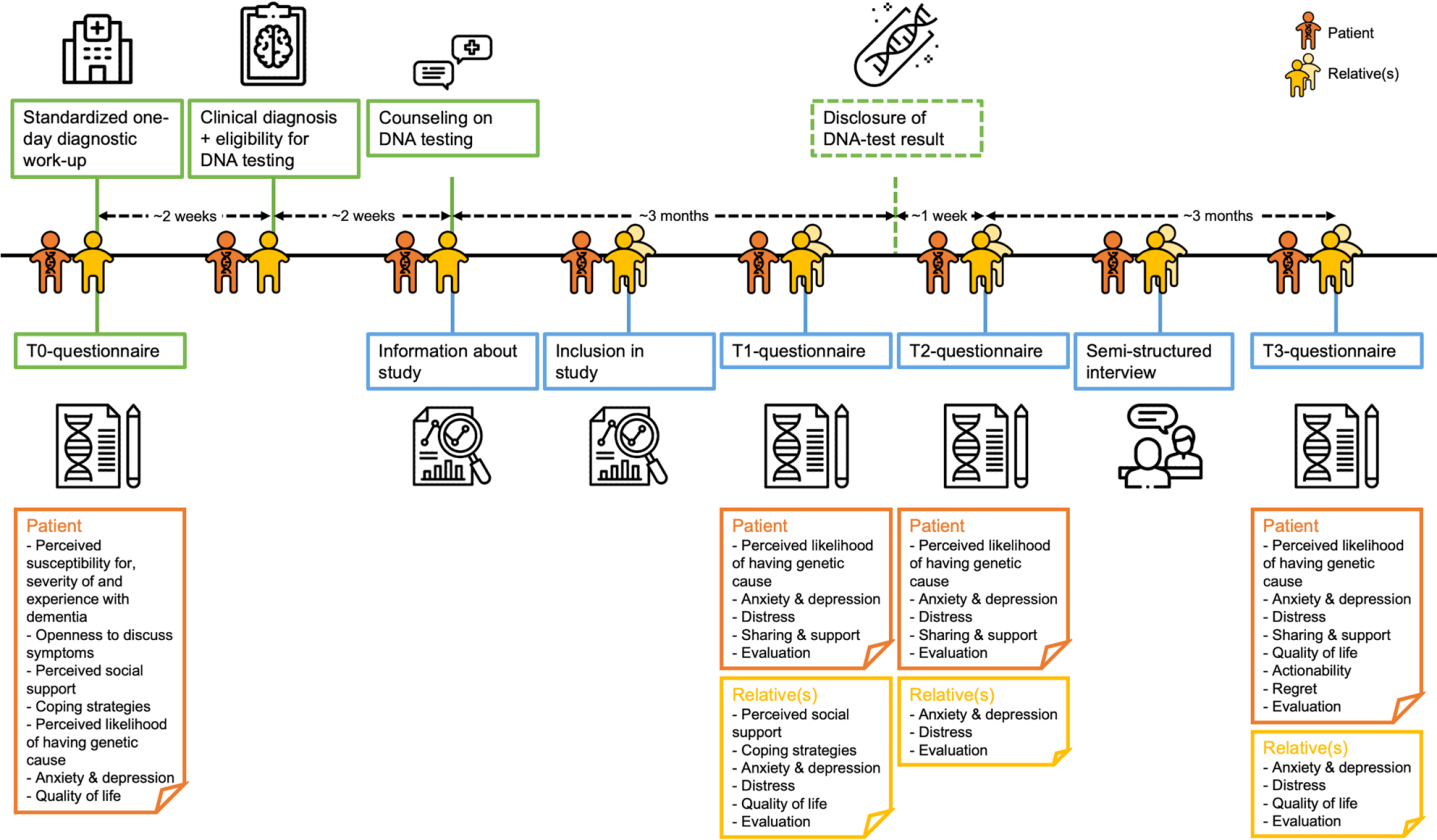
Timeline of participants' visits and assessments. Green blocks represent procedures of standard care in Alzheimer Center Amsterdam, blue blocks represent procedures of this study regarding impact. Orange persons and questionnaires pertain to patients, yellow persons and questionnaires pertain to relatives. The following measures were used to assess psychological and psychosocial and behavioral factors: Perceived susceptibility for, severity of and experience with dementia, assessed by first two subscales and an added third of the Motivation to Change Lifestyle and Health Behaviors for Dementia Risk Reduction Scale (MCLHB-DRR) [19, 20]; Openness to discuss symptoms in the family, assessed by adapted version of Openness to Discuss Cancer in the Family (ODCF) scale [21, 22]; Perceived social support, assessed by Multidimensional Scale of Perceived Social Support (MSPSS) [23]; Coping strategies, assessed by BRIEF-COPE scale [24]; Perceived likelihood of genetic cause, assessed by a visual analogue scale (VAS) with a 0-100 range; Anxiety and depression, assessed by Hospital Anxiety and Depression Scale (HADS) [25]; Distress, assessed by Impact of event (IES) [26]; Quality of life, assessed by Brunnsviken Brief Quality of Life Scale (BBQ) [27] and a VAS with a 0–10 range; Actionability, assessed by a self-developed five-item scale derived from relevant literature (Supplement 2); Sharing with and support from partner, family and others, assessed by a self-developed five-item scale (Supplement 2); Decision Regret Scale, assessed by Decision Regret Scale (DRS) [28]; Satisfaction with counseling, disclosure and the overall experience, each assessed by a VAS with a 0–10 range, and text fields for optional comments. Expanded version of the study design previously published in [16], now showing all assessment time points

Patients eligible for monogenic testing were identified based on data-driven criteria [15]. Their interest and considerations in deciding to (not) pursue this option have been reported elsewhere [16]. Here, we focus on the impact of disclosing the test results to patients and their relatives. As shown in Fig. 1, at their first visit (T0) patients underwent a standardized one-day diagnostic work-up consisting of medical, neurological, and neuropsychological assessment, magnetic resonance imaging (MRI) and optional cerebrospinal fluid (CSF) analysis. Demographic and medical data were retrieved from their medical records. Additionally, they filled out a questionnaire assessing psychosocial characteristics, including perceived susceptibility for, severity of and experience with dementia, openness to discuss symptoms in the family, perceived social support, coping strategies, as well as baseline measures of outcomes, including perceived likelihood of having a genetic cause, anxiety, depression, and quality of life.

In a multi-disciplinary meeting, the clinical diagnosis and eligibility for monogenic testing were established, which were conveyed to patients and their caregivers by their treating neurologist. In case of a highly suspicious family history, they were referred to a geneticist for additional counseling. Patients and / or one or more relative(s) were invited to this study, and after providing written informed consent, participants completed additional questionnaires assessing psychosocial and behavioral outcome measures, including perceived likelihood of having a genetic cause, anxiety, depression, distress, quality of life, actionability, regret as well as their evaluation of the counseling, disclosure process, and overall experience. These were administered one week after counseling (T1), as well as one week (T2) and three months (T3) after disclosure. Patients completed these independently, with assistance from a relative, or via proxy when necessary, and this mode of completion was recorded for each questionnaire. When patients were no longer willing or able to complete the assessments due to cognitive impairment or study-related burden, we discontinued their participation in consultation with the patient and/or relative.

### Measures

Perceived susceptibility for, severity of, and experience with dementia, were assessed using the first two subscales and an added third of the Motivation to Change Lifestyle and Health Behaviors for Dementia Risk Reduction Scale (MCLHB-DRR) [19, 20]. Openness to discuss symptoms in the family was assessed with an adapted version of Openness to Discuss Cancer in the Family (ODCF) scale [21, 22]. Perceived social support was assessed using the Multidimensional Scale of Perceived Social Support (MSPSS) [23]. Coping strategies were assessed with the BRIEF-COPE scale [24]. Perceived likelihood of carrying a genetic cause was assessed by a visual analogue scale (VAS). Anxiety and depression were assessed with the Hospital Anxiety and Depression Scale (HADS) [25]. Distress was assessed using the Impact of Event Scale (IES) [26]. Quality of life was assessed via the Brunnsviken Brief Quality of Life Scale (BBQ) [27] and a VAS. Actionability was assessed using a self-developed five-item Likert-scale derived from relevant literature, addressing seeking support, preparing for the future, changing plans or priorities, adjusting lifestyle, and registering for research participation. Sharing with and support from partner, family and others were assessed using a self-developed five-item Likert-scale. Decision regret was assessed with the Decision Regret Scale (DRS) [28]. Finally, satisfaction with counseling, disclosure and the overall experience were each assessed through a VAS, supplemented with optional text fields for additional comments.

### Interviews

To further explore their experiences regarding monogenic testing, participants were invited to participate in optional semi-structured in-depth interviews. These lasted 45–60 min and, depending on participants’ preferences, were conducted with either the patient, a relative or both jointly, and took place at home, digitally via Microsoft Teams or in person. Topics included comprehension of, reactions to, and implications of the test results (see *Supplement 1* for topic guide).

### Quantitative analysis

When questionnaires were incomplete, we only included scales with all items answered in the analysis. Differences in psychosocial factors between groups were analyzed using one-way ANOVA, Kruskal-Wallis, Pearson’s χ², or Fisher’s Exact Test, depending on data type and distribution. We used linear mixed models to calculate effects of group (negative or positive test result), time, and interactions on outcome variables. Data from patients and relatives were analyzed separately. Effects with p-values < 0.05 were considered significant and are reported. Due to the exploratory nature of our aims, no adjustment for multiple comparisons were performed. All analyses were executed using the statistical software R version 4.3.2 (R Foundation for Statistical Computing).

### Qualitative analysis

JS conducted all interviews and audio recordings were transcribed verbatim. We followed an inductive approach to allow concepts to emerge directly from the participants’ narratives [29]. JS and EA examined the content to generate descriptive codes, which were then iteratively refined and organized into broader categories to capture the richness and complexity of experiences. The resulting coding tree was discussed in regular meetings between JS and EA. To enhance the reliability and validity, three interviews were independently coded by JS and EA. Discrepancies were discussed until consensus was reached on the majority of codes applied to the same segments. The finalized coding scheme was then used by JS to code all remaining interviews. Based on the frequency, coherence, and relevance of codes across the dataset, JS identified overarching themes, which were reviewed and refined in multiple rounds with input from EA, WF, and LV. Data were managed using MAXQDA-software version 22.1.1, and results are reported in accordance with the Consolidated Criteria for Reporting Qualitative Research (COREQ) guidelines [30].

## Results

Between October 18, 2021, and October 26, 2022, we examined 31 patient cases visiting Alzheimer Center Amsterdam who met eligibility criteria for genetic testing for monogenic causes and decided to pursue this option. Of these, 25 tested negative and six positive (*AARS2*, *HTRA1*, *NOTCH3*, *MAPT*, *PSEN2* and a variant of uncertain significance [VUS]). In total, we included 26 patients and 29 relatives (in 3/31 cases we included only patients, in 5/31 only relatives and in the 23/31 remaining cases both).

### Characteristics of patients and relatives

The demographic, clinical and psychosocial characteristics of the patient cases (*n* = 31) at first visit (T0) are shown in Table 1. Less than half were female (14/31, 45%) and average age at presentation was relatively young (61 ± 8 years). Half reported a family history of dementia (16/31, 52%) and the majority had children (27/31, 87%). They perceived the likelihood of having a genetic cause to be 43% (sd = 36%). When comparing groups, differences in distribution of diagnoses (*p* < 0.05), and social support (6.3 ± 0.7 vs. 5.5 ± 1.0, *p* < 0.05) were observed, with patients who tested positive being less often clinically diagnosed with AD and reporting lower levels of social support. No other demographic, medical, or psychosocial factors were related to the presence of a genetic cause prior to testing.

**Table 1:** Demographic, medical and psychosocial characteristics of patient cases at first visit (T0), stratified by group (negative / positive)

	all (*n* = 31)	negative (*n* = 25)	positive (*n* = 6)	*p*-value
Sex (*n* (%) female)	14 (45%)	12 (48%)	2 (33%)	0.66
Age (mean ± sd years)	61 ± 8	61 ± 8	59 ± 9	0.62
Education (mean ± sd)	5 ± 1	5 ± 1	5 ± 1	0.52
Has children (*n* (% yes))	27 (87%)	23 (92%)	4 (67%)	0.16
Geographical origin				0.72
European (*n* (%))	22 (71%)	18 (72%)	4 (67%)	
Moroccan (*n* (%))	2 (6%)	2 (8%)	0 (0%)	
Turkish (*n* (%))	0 (0%)	0 (0%)	0 (0%)	
Other (*n* (%))	2 (6%)	1 (4%)	1 (17%)	
Not reported (*n* (%))	5 (16%)	4 (16%)	1 (17%)	
Has family history (*n* (% yes))	16 (52%)	12 (48%)	4 (67%)	0.65
MMSE (mean ± sd)	23 ± 5	23 ± 5	24 ± 7	0.51
Diagnosis				0.02 *
SCD (*n* (%))	1 (3%)	0 (0%)	1 (17%)	
MCI (*n* (%))	1 (3%)	0 (0%)	1 (17%)	
Dementia (*n* (%))	25 (81%)	22 (88%)	3 (50%)	
AD (*n*)	15	14	1	
FTD (*n*)	2	2	0	
VaD (*N*)	1	0	1	
DLB	1	1	0	
PPA	4	4	0	
Other (*n*)	2	1	1	
Other/undetermined (*n* (%))	4 (13%)	3 (12%)	1 (17%)	
Perceived susceptibility for dementia (3–15) (mean ± sd)	11 ± 3	10 ± 3	9 ± 3	0.08
Perceived severity of dementia (5–25) (mean ± sd)	15 ± 5	16 ± 5	14 ± 6	0.68
Experience with dementia (2–10) (mean ± sd)	6 ± 2	6 ± 2	7 ± 3	0.40
Perceived likelihood of genetic cause (%±sd)	43 ± 36	43 ± 36	43 ± 36	0.99
Openness to discuss symptoms in family (9–45) (mean ± sd)	34 ± 6	35 ± 6	32 ± 4	0.25
Perceived social support (1–7) (mean ± sd)	6.1 ± 0.8	6.3 ± 0.7	5.5 ± 1.0	0.04 *
Coping strategy				
Problem-focused (1–4) (mean ± sd)	2.6 ± 0.7	2.7 ± 0.7	2.2 ± 0.8	0.11
Emotion-focused (1–4) (mean ± sd)	2.3 ± 0.5	2.5 ± 0.5	2.0 ± 0.4	0.07
Avoidant (1–4) (mean ± sd)	1.8 ± 0.5	1.9 ± 0.5	1.6 ± 0.3	0.25
Anxiety (0–21) (mean ± sd)	6.5 ± 4.9	6.9 ± 5.2	5.0 ± 4.0	0.41
Depression (0–21) (mean ± sd)	4.5 ± 3.6	4.8 ± 3.7	3.0 ± 2.8	0.27
Quality of life				
BBQ (0–96) (mean ± sd)	53 ± 19	55 ± 18	47 ± 21	0.38
VAS (0–10) (mean ± sd)	7.5 ± 1.5	7.3 ± 1.5	8.4 ± 0.9	0.10

*AD* Alzheimer’s Disease, *BBQ* Brunnsviken Brief Quality of Life Scale, *DLB* Dementia with Lewy bodies, *FTD* Frontotemporal Dementia, *MCI* Mild Cognitive Impairment, *MMSE* Mini-Mental State Examination, *PPA* Primary Progressive Aphasia, *SCD* Subjective Cognitive Decline, *VaD* Vascular Dementia, *VAS* visual analog scale

Education was rated using the Dutch Verhage system (range: 1-7). Other instruments are listed in Fig. 1

* We found group differences in distribution of diagnosis and perceived social support (*p*< 0.05)

Comparison between patients’ decisions were calculated with Pearson’s χ², Mann-Whitney U or Independent Samples t-tests

The demographic and psychosocial characteristics of the relatives (*n* = 29) after counseling (T1) are shown in Table 2. More than half were female (16/29, 55%) and average age was 54 ± 12 years. Most were partners (17/29, 59%), followed by children (8/29, 28%) and siblings (4/29, 14%). Relatives of patients who tested negative versus positive were comparable.

**Table 2:** Demographic, and psychosocial characteristics of relatives after counseling (T1), stratified by group (negative / positive)

	all (*n* = 29)	negative (*n* = 24)	positive (*n* = 5)	*p*-value
Sex (*n* (%) female)	16 (55%)	12 (50%)	4 (80%)	0.34
Age (mean ± sd years)	54 ± 12	55 ± 12	51 ± 14	0.58
Relationship with patient				0.22
Partner	17 (59%)	15 (62%)	2 (40%)	
Sibling	4 (14)	2 (8%)	2 (40%)	
Child	8 (28%)	7 (29%)	1 (20%)	
Education (mean ± sd)	6 ± 1	6 ± 1	6 ± 1	0.30
Perceived social support (1–7) (mean ± sd)	5.8 ± 1.0	5.8 ± 1.1	5.6 ± 0.5	0.57
Coping strategy				
Problem-focused (1–4) (mean ± sd)	2.7 ± 0.5	2.7 ± 0.6	2.9 ± 0.4	0.61
Emotion-focused (1–4) (mean ± sd)	2.4 ± 0.4	2.4 ± 0.5	2.4 ± 0.3	0.96
Avoidant (1–4) (mean ± sd)	1.6 ± 0.4	1.6 ± 0.4	1.5 ± 0.2	0.40
Anxiety (0–21) (mean ± sd)	6.8 ± 4.4	6.7 ± 4.6	7.4 ± 4.0	0.74
Depression (0–21) (mean ± sd)	4.4 ± 4.0	4.5 ± 4.1	4.4 ± 3.9	0.98
Distress ((mean ± sd)	16 ± 17	15 ± 17	22 ± 20	0.44
Quality of life				
BBQ (0–96) (mean ± sd)	60 ± 23	59 ± 24	63 ± 18	0.76
VAS (0–10) (mean ± sd)	7.6 ± 1.5	7.5 ± 1.6	8.0 ± 1.2	0.55

*BBQ* Brunnsviken Brief Quality of Life Scale, *VAS* visual analog scale

Education was rated using the Dutch Verhage system (range: 1-7). Other instruments are listed in Fig. 1

Comparison between patients’ decisions were calculated with Pearson’s χ², Mann-Whitney U or Independent Samples t-tests.

Patients completed 95 questionnaires, independently in 38 (40%), with assistance from a relative in 48 (51%), and by proxy in 9 (9%). Relatives completed 75 questionnaires. Education was rated using the Dutch Verhage system (range: 1–7). Other instruments are listed in Fig. 1.

### Psychosocial and behavioral outcomes

**Fig. 2:**
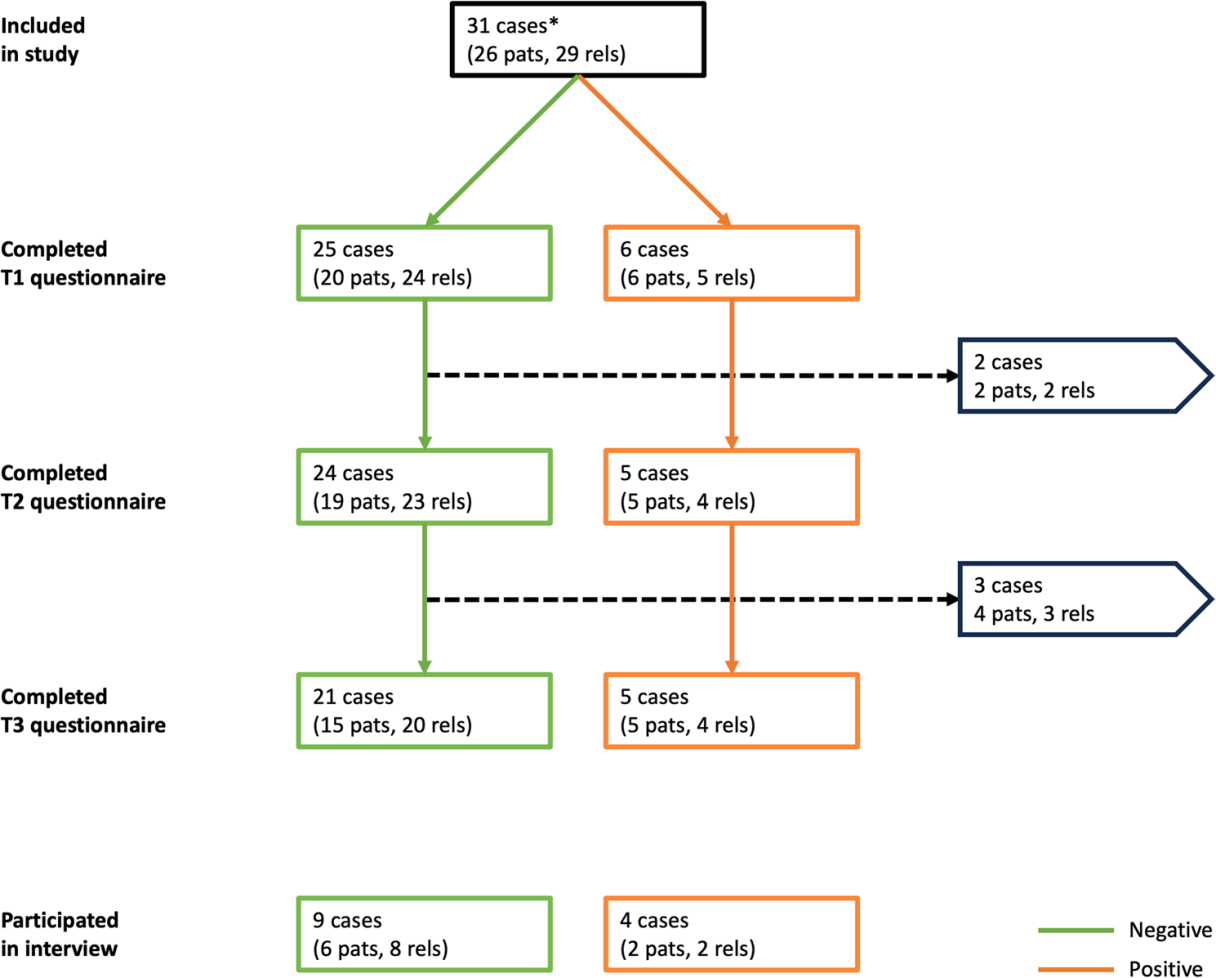
Flow diagram of study participants * A case refers to the symptomatic individual undergoing genetic testing for monogenic causes of dementia. For each case, either the patient, one or more relatives, or both were included in the study (specifically, in three cases only patients were included, in five only relatives, and in the remaining 23 cases both). Only patients were offered genetic testing, relatives were only invited to fill out questionnaires and participate in interviews Abbreviations: pats, patients; rels, relatives

As shown in Fig. 2, the full set of three questionnaires was completed by 20 patients and 24 relatives related to 26 cases (21 tested negative and 5 positive). Six patients and five relatives discontinued participation due to the burden of the disease, the study or both. In addition, we conducted 13 semi-structured interviews (three with patients, five with relatives, and five with both). These pertained to nine negative and four positive cases (including a variant of uncertain significance).

Results are presented in a mixed-methods format, beginning with quantitative outcomes from the questionnaires, followed by qualitative findings from the interviews to provide context and deepen understanding.

#### Comprehension of test result

**Fig. 3:**
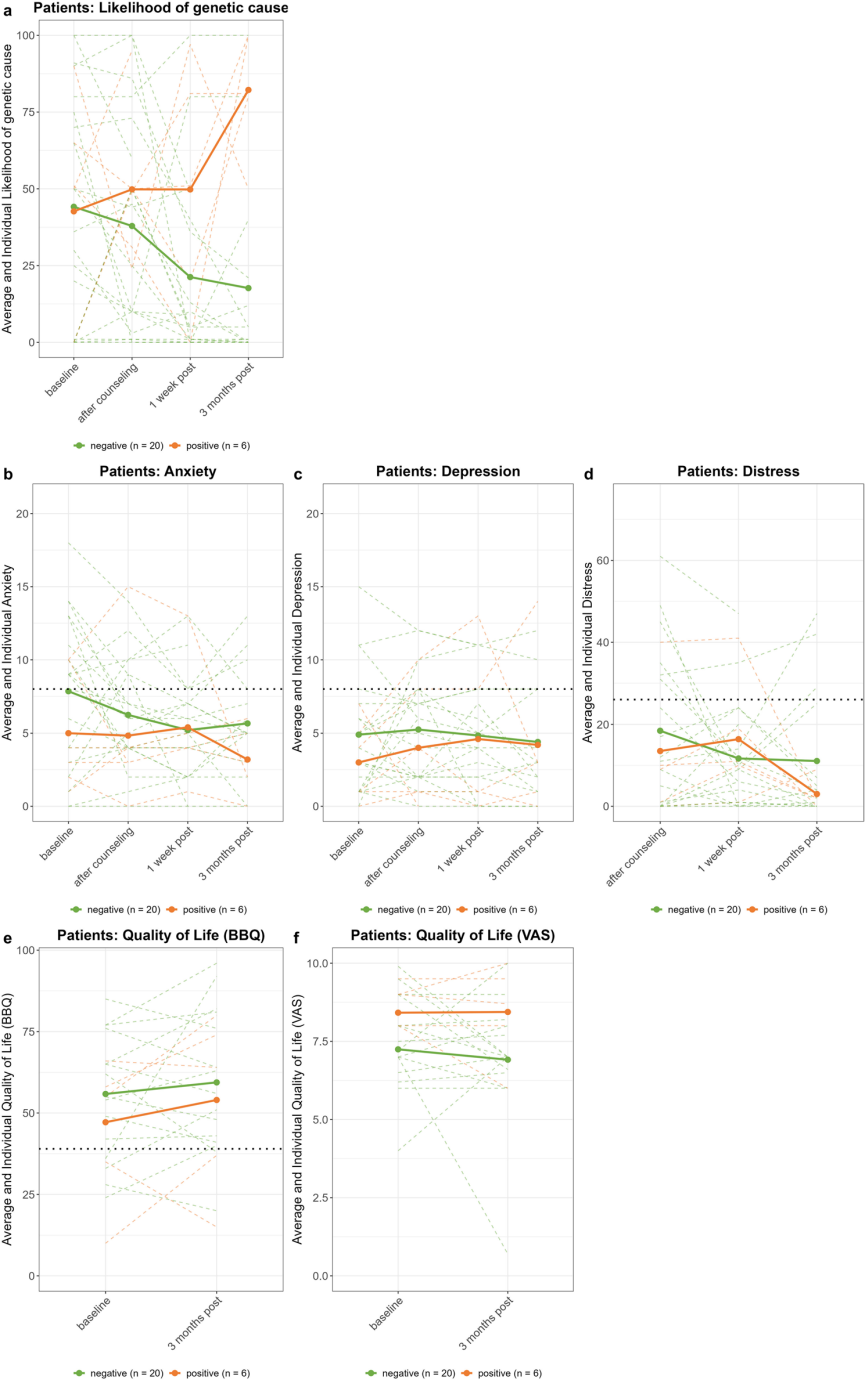
Comprehension and psychological impact on patients. **a** Patients’ perceived likelihood of a genetic cause. **b-f** Patients' psychological impact. Solid colored lines represent average scores per group, dotted colored lines denote individual scores. Black horizontal dotted lines indicate cut-off for clinical significance of anxiety, depression and distress BBQ = Brunnsviken Brief Quality of Life Scale, VAS = visual analogue scale

As Fig. 3a shows, perceived likelihood of a genetic cause was lower in patients who tested negative at one week (95% CI [–37.33, − 5.90], *p* < 0.01) and three months post-disclosure (95% CI [–40.79, − 6.82], *p* < 0.01) compared to baseline, reflecting a main effect of time. At three months, patients who tested positive reported higher scores compared to those who tested negative (95% CI [32.11, 101.07], *p* < 0.001), indicating a significant time × result interaction.

In interviews, participants often spoke in general terms about clinical testing, not clearly distinguishing the genetic component. However, when specifically asked about the latter, most participants demonstrated an adequate understanding of a negative result, ranging from recognizing the disease was not “hereditary”, or “due to a genetic cause”, to grasping that: “most known [genes] were ruled out and a small element of uncertainty remains” (patient, case 110039, negative). In contrast, a positive test result was equated to “an inherited form” of dementia, “meaning if you have it, you will definitely develop [dementia]” (patient, case 110027, positive). One patient described his VUS as: “One gene that wasn’t entirely right […] and they don’t exactly know what it does or means.” (patient, case 110031, VUS).

#### Psychological impact

**Fig. 4:**
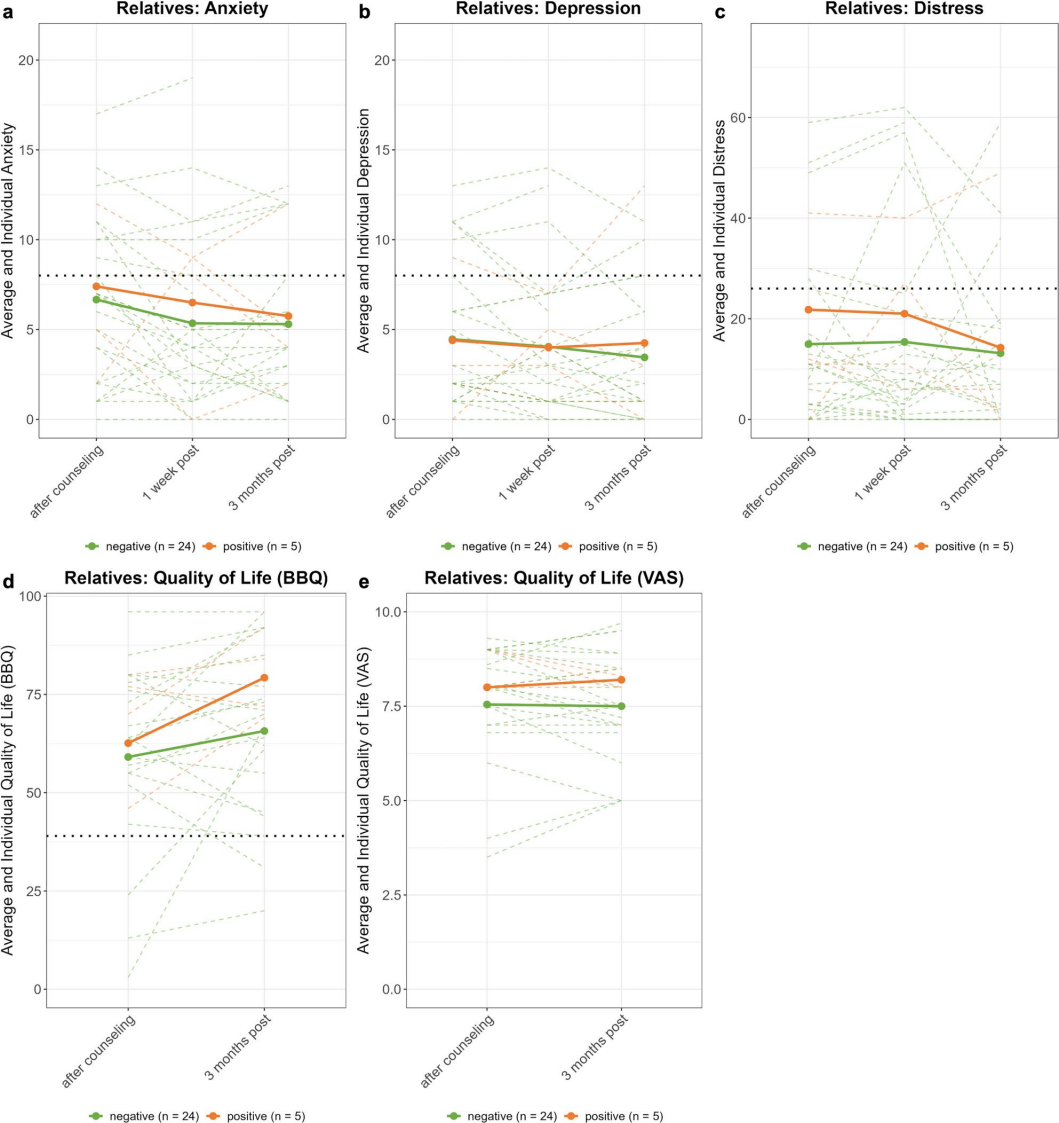
Psychological impact on relatives. Solid colored lines represent average scores per group, dotted colored lines denote individual scores. Black horizontal dotted lines indicate cut-off for clinical significance of anxiety, depression and distress. BBQ = Brunnsviken Brief Quality of Life Scale, VAS = visual analogue scale.

As Figs. 3b-f and 4 show, average levels of anxiety, depression, and distress remained below the clinical threshold, while quality of life scores were well above the cutoff. Across groups, anxiety was lower at one week (95% CI [–4.37, − 0.71], *p* < 0.01) and three months post-disclosure (95% CI [–4.14, − 0.18], *p* < 0.05; Fig. 3b) compared to baseline, reflecting a main effect of time. Likewise, in relatives, anxiety was lower at one week post-disclosure (95% CI [–2.61, − 0.14], *p* < 0.05; Fig. 4a), but not at three months. We observed no other effects of test result, time point, or their interaction, in patients or relatives.

In interviews, most participants noted genetic testing did not directly affect the patient: “He already has dementia, that doesn’t change” (relative, case 110019, negative). However, after receiving negative results they reported feelings of relief, happiness or elation, knowing their (grand)children were not at risk of having inherited a pathogenic mutation, even though they realized this didn’t safeguard their offspring from developing sporadic dementia. Several patients felt unburdened by worries, responsibility or guilt: “It’s a very reassuring thought. They may still develop [dementia], but not due to my DNA.” (patient, case 110012, negative). Nevertheless, some experienced ambivalence, questioning how the disease had originated instead. By contrast, when a genetic cause was identified, participants often perceived this as a confirmation of their fears: “You already know you have to deal with [the disease], and when it occurs so often you feel there must be something in the family. So it didn’t come as a bolt from the blue.” (patient, case 110027, positive). In children, however, the test result elicited fear and uncertainty about “having a 50% chance of also getting this [disease] and ending up like your [parent]” (relative, case 110009, positive). Still, they appreciated clarity about what affected their families.

#### Communication and support

**Fig. 5:**
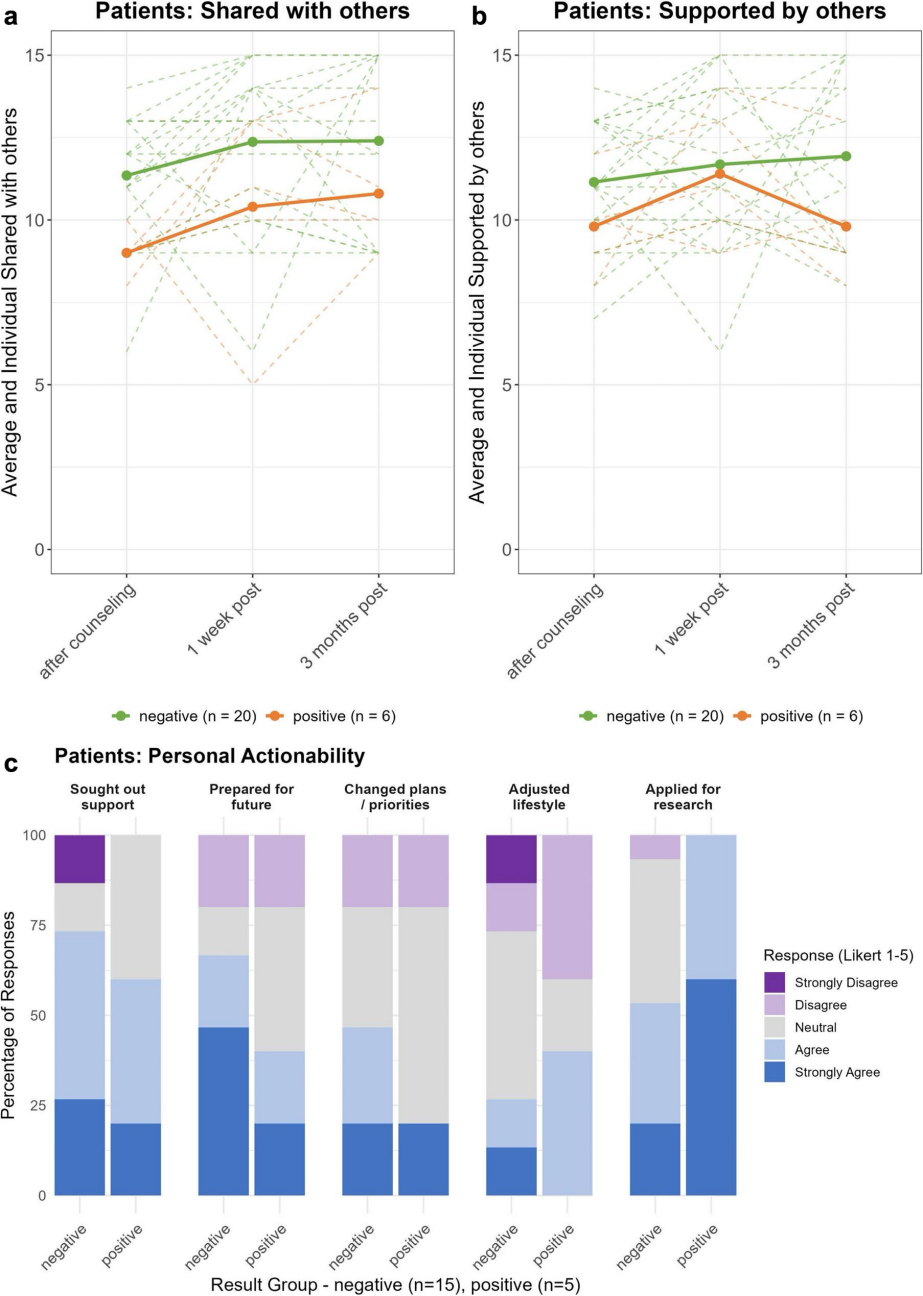
Communication and personal actionability. **a** Extent to which patients discussed genetic testing and the result with partners, families and others. **b** Extent to which patients felt supported by partners, families and others regarding genetic testing and the result. Solid colored lines represent average scores per group, dotted colored lines denote individual scores. **c** Patients' personal actionability

As Fig. 5a-b shows, patients who tested positive discussed the possibility of a monogenic cause less often with partners, families and others compared to those who tested negative (95% CI [–4.65, − 0.05], *p* < 0.05), irrespective of time. Support from partners, family, and others showed no significant effects of time, group, or their interaction.

In interviews, most participants reported having discussed genetic testing and shared their results with adult first-degree relatives, expecting them to inform more distant family members. Beyond this, some spoke about it openly with friends and acquaintances, processing events, seeking support or seeing no reason to withhold it: “I’ve never been worse off for putting everything on the table, it’s never been used against me, so that’s my way of dealing with difficult things: talking them through.” (relative, case110019, negative).” However, most conveyed the news only selectively or not at all, reluctant to reveal the diagnosis or the test result for fear of stigma: “Once you know someone has Alzheimer’s or dementia, you see them differently” (patient, case 110012, negative). Others viewed it as a family matter, irrelevant to others, or no longer topical in the absence of a genetic cause: “Once we got the result, it didn’t really come up again.” (relative, case 11001, negative).

While perceived support did not differ significantly between groups, only participants related to positive cases described challenges in receiving support from partners, family members, or others. Some relatives experienced emotional distance from their siblings, which they attributed to differences in coping strategies and the caregiving burden associated with their affected family member. These dynamics often left little space to support one another and contributed to feelings of isolation. Another reflected on the limitations of social support more broadly: “There are times when I feel unsupported by some friends. But at the same time, I know I have to go through this on my own. They can help me by listening to my story, but no one can take this away from me.” (relative, case 110009, positive).

#### Personal actionability

As Fig. 5c shows, three months after receiving their test results, most patients reported seeking support, making preparations for the future, or registering for research participation. A minority did so for changing their lifestyle or adjusting plans or priorities. Those who tested positive more often registered for research participation compared to those who tested negative (*W* = 15.5, *p* < 0.05). No other differences in responses to potential actions were observed.

In interviews, participants clarified that any personal utility primarily stemmed from the syndrome diagnosis rather than genetic testing, except for research participation. Among those who tested positive, a patient intended to enroll in a secondary prevention trial, while a relative hoped to receive disease-specific guidance and support, but expressed disappointment about the extensive data shared with researchers and the little information returned by them: “What is the point of knowing he has that specific type [of dementia], if it doesn’t lead to a different approach?” (relative, case 110016, positive). Instead, participants agreed genetic testing offered more personal actionability or medical utility for at-risk relatives, including decisions on presymptomatic testing, reproductive intentions, life planning, therapeutic treatments, lifestyle interventions, financial preparation, timely diagnosis or appropriate support.

#### Decision regret

**Fig. 6:**
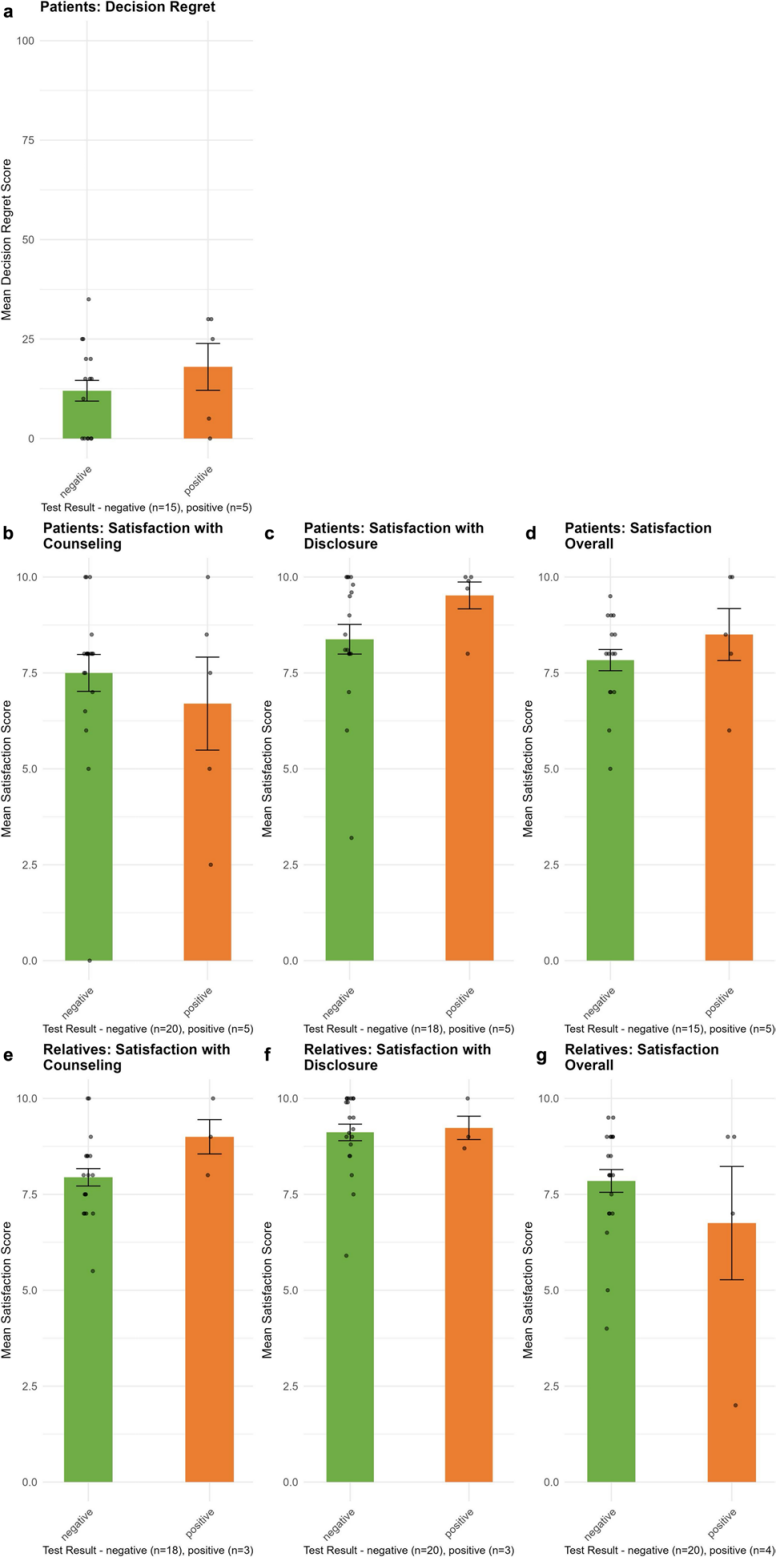
Decision regret and satisfaction. **a** Patients’ decision regret three months after disclosure. **b**-**g** Patients’ and relatives’ satisfaction.

As Fig. 6a shows, patients’ decision regret was low and independent of group.

When asked in interviews about their advice for other patients presented with the option of genetic testing, some participants recommended that it should always be pursued, citing its potential to contribute to research or benefit relatives. Others emphasized that the decision is highly personal and contingent on their circumstances: “It completely depends on the kind of people they are.” (relative, case 110019, negative). Among those who tested positive, one participant stressed the importance of considering in advance how the result might affect their family, and where to seek support. Another advised against testing in the absence of cognitive symptoms, stating: “If you know this is hanging over you, it can definitely affect your mindset. I don’t think that’s a good thing, because you start doing things differently that you otherwise wouldn’t. You begin anticipating it already, while that may be completely unnecessary, because it might not manifest until twenty years from now.” (patient, case 110027, positive).

#### Satisfaction with care

As Fig. 6b-g shows, patients and relatives were satisfied with counseling, disclosure and the overall experience with no differences between groups. Still, 28% (9/32) of patients and 21% (7/34) of relatives indicated a need for additional information, which did not vary by group. Questions related to DNA-diagnostics in general, the impact of the test result on their prognosis, how to inform their family of a genetic cause, implications for at-risk relatives, and predictive genetic testing. One relative expressed a desire for more knowledge about genetic testing and its implications, noting that they had been inadequately informed by their family members (relative, case 110036, negative).

In interviews, several participants shared that they initially viewed counseling as unnecessary or even paternalistic but came to appreciate it in retrospect. Some suggested providing supplementary information, including audio recordings, textual summaries, or visual representations, to better accommodate patients, to enable revisiting the content, or to facilitate sharing it with relatives.

## Discussion

This mixed-methods study aimed to explore the psychosocial and behavioral consequences of diagnostic testing for monogenic causes of dementia in patients and their families. While quantitative findings showed no evidence of psychosocial harm, the qualitative data revealed a more complex picture. Our findings show contrasting and cascading emotional, relational and practical implications that extended beyond patients and rippled through families. This was first evident in the immediate response. A negative result often brought relief and reassurance that children were not at risk, yet sometimes left uncertainty about what had triggered early-onset symptoms of cognitive decline. In contrast, a positive result frequently evoked worry and fear for relatives, while offering clarity about the inherited origin of dementia. Social interactions further reflected this pattern: in cases with a confirmed monogenic cause, patients reported discussing this less often with family and friends, while relatives described challenges in finding support. This dynamic also emerged in the third domain, as apart from research participation, personal actionability was considered more pertinent for at-risk relatives. Nevertheless, participants valued genetic testing and reported no regret.

Despite (subjective) cognitive decline in all patients and a clinical diagnosis of dementia in most, both questionnaires and interviews indicated that they were able to grasp the basic meaning of their genetic status. This is consistent with findings from a study in a memory clinic setting, where the majority of participants accurately recalled their result six months after disclosure, although outcomes were reported by patient–relative dyads [14]. Survey data from individuals with amyotrophic lateral sclerosis (ALS) also indicate that most found the information understandable [31], with the caveat that this population likely differed from our cohort with respect to cognitive impairment. Taken together, these observations support the plausibility that, in this setting, participants could generally understand the core meaning of their genetic status.

Throughout the process, average levels of psychological outcomes remained below the clinical threshold in patients and relatives, with anxiety decreasing after disclosure, and no differences between those with a negative versus a positive result. However, comparative data are scarce. While presymptomatic testing has been studied more extensively, the psychosocial impact of symptomatic testing for monogenic causes of dementia has received little attention. This lack of research on the proband (i.e., the first clinically affected individual in a family to be evaluated for a genetic cause) is not unique to dominantly inherited neurodegenerative diseases, but has also been reported as a critical “oversight” in the field of oncology [32–34]. Few studies have examined diagnostic testing in memory clinic populations [14, 35, 36], and to our knowledge, none reported quantitative psychological outcomes both before and after testing. However, one study using a similar design in patients with suspected Huntington’s disease (HD) found no differences in psychological outcomes between patients with or without a confirmed genetic cause [37], aligning with our findings. In contrast, research involving individuals tested at different intervals after being diagnosed with breast or ovarian cancer showed differing responses, with significantly greater distress among those assessed within the first year [38–40], suggesting the timing of the genetic testing may be associated with the psychosocial impact of receiving the result. As such, the post-disclosure declines in anxiety we observed in patients and relatives, regardless of test result, may reflect a broader relief associated with moving from uncertainty to diagnostic clarity. Alternatively, it may be attributable to the predominance of negative results, with the number of positive cases likely too small to detect a statistically meaningful difference. These findings emphasize the need to address the evidence gap on the impact of diagnostic testing.

The interview data provided additional context to these findings, illustrating how participants perceived the emotional impact of genetic testing to be shaped primarily by its relevance to their families. Reactions, whether of relief or distress, were largely directed toward the implications for (grand)children and other at-risk family members. This perspective is reinforced by relatives’ own accounts, with some describing intense emotional responses centered on the implications for themselves, particularly when a genetic cause had been identified. However, we found no prior research examining how diagnostic genetic testing in patients affects the immediate psychological well-being of their relatives, in the context of dementia, other neurodegenerative disorders, or cancer syndromes with dominant inheritance. Nevertheless, research on longer-term implications suggests “living at risk” for familial neurodegenerative disorders, can carry substantial psychological and relational burden, indicating that identifying a monogenic cause may have significant downstream effects on relatives [41, 42]. Future studies should assess how receiving a patient’s genetic result affects the well-being of family members.

These observations may be all the more significant in light of the limited social support reported by both patients and their relatives. Even at the first visit, patients who later received a positive result reported lower levels of perceived social support. Throughout the testing trajectory, they discussed the topic less often with partners, family, and others. Following disclosure, most participants reported conveying the results to adult first-degree relatives, expecting them to inform more distant family members. Some relatives of patients with a confirmed genetic cause described emotional distance, feelings of isolation, and challenges in finding support both within and beyond the family. A retrospective study on diagnostic genetic testing for ALS or FTD suggests such difficulties may persist over time, with participants describing barriers to sharing information, postponed disclosure to at-risk children, processing such news in isolation, and with little access to support, contributing to lasting effects on family communication and dynamics [36]. A similar study on diagnostic genetic testing for younger-onset dementia described how these challenges may be further compounded by pre-existing tensions, arising from the emotional burden of witnessing a loved one’s decline, unequal caregiving responsibilities, conflicting views on care, or divergent coping styles [43]. These findings underscore the need for psychosocial support to help families navigate the challenges of living with the knowledge of a confirmed genetic cause.

Participants generally agreed that testing for monogenic causes of dementia offered greater personal actionability and medical utility for at-risk relatives than for patients. Among the five aspects assessed three months after disclosure, having registered for research participation was the most strongly endorsed behavioral response, with a higher proportion among patients who tested positive compared to those who tested negative (100% versus 53%). In contrast, lifestyle changes were reported by 30% (independent of test result). This pattern aligns with findings in patients with cognitive impairment who had undergone genetic testing for familial dementia, where 36% indicated they were (very) likely to make health or wellness changes after disclosure, yet only 31% reported having done so six months later [14]. In this context, contributing to research offers patients a purposeful way to help their at-risk family members.

Lastly, we found participants were satisfied with counseling, disclosure, and the overall experience, with no differences between groups. In addition, patients’ decision regret was low and unrelated to the test result. These findings are consistent with a prospective study on diagnostic genetic testing in a memory clinic population [14] as well as a retrospective survey in patients with ALS [31]. Nonetheless, 28% of patients and 21% of relatives indicated a need for additional information, irrespective of test result, highlighting the importance of supplementary materials and follow-up information to enhance clarity and address remaining questions.

### Strengths and limitations

A key strength of this study is its real-world clinical setting, involving patients for whom genetic testing for monogenic causes of dementia was warranted. All participants were seen at a tertiary memory center, underwent a standardized diagnostic work-up, with eligibility for testing determined by data-driven clinical criteria. By starting data collection at the first visit, before genetic testing was offered, and continuing until three months after disclosure, we were able to capture a rare and comprehensive overview of patient and family experiences throughout the diagnostic trajectory. In addition, in-depth interviews enriched findings from standardized questionnaires by contextualizing individual responses and revealing nuances not captured by scales. Furthermore, the inclusion of relatives allowed for examination of the broader psychosocial impact of identifying monogenic causes for familial dementia, addressing a persistent gap in literature.

The sample size of this study was modest, reflecting the inherent challenges of recruiting individuals undergoing diagnostic evaluation for dementia. Many patients and relatives were confronted with the impact of the diagnosis, disease progression, and the demands of care, which limited participation. In addition, the rarity of monogenic causes, identified in 3.3% of memory clinic populations [15], further constrained the eligible pool. Nevertheless, we were able to follow 31 cases longitudinally, providing valuable insight into the experiences of patients and relatives throughout the diagnostic process. However, the small number of participants with a positive result limited between-group comparisons, and the absence of adjustment for multiple testing increases the likelihood of chance findings. These exploratory findings should therefore be interpreted cautiously and validated in larger studies.

A further limitation concerns patients’ understanding of their genetic test result in the context of cognitive impairment. As we did not formally assess comprehension or recall using a validated instrument, we cannot draw firm conclusions in this regard. In more than half of cases, questionnaires were completed with assistance from a relative, which may have facilitated understanding and informed participation. This is consistent with prior memory-clinic work suggesting that involvement of a cognitively intact co-participant can help ensure informed engagement with genetic testing and its implications [14]. At the same time, such support may also shape how questions are interpreted and answered, meaning that some responses may reflect a shared perspective rather than the patient’s views alone. Future research should include direct assessment of comprehension and recall in cognitively impaired patients and explicitly distinguish patient-only from assisted or proxy responses.

Lastly, the demographic composition of our sample reflects the patient population typically seen in our memory clinic, comprising predominantly younger, highly educated individuals of White descent. This highlights the importance of future research efforts aimed at including individuals from underrepresented groups, particularly those from varied racial, ethnic, socioeconomic, and educational backgrounds. Broadening participation is essential to improve the generalizability of findings and to inform the development of equitable, culturally responsive care and support systems for families facing familial dementia.

## Conclusion

This study demonstrates that quantitative findings indicated no substantial psychosocial harm, with qualitative insights adding important nuance by showing complex emotional, relational, and practical implications that extend beyond the individual and affect families as a whole. Particularly when a genetic cause was confirmed, families described difficulties in accessing support, highlighting a clear need for tailored psychosocial care. Despite these challenges, participants valued the opportunity for genetic testing and expressed no regret. Taken together, these insights support the careful integration of genetic testing into memory clinic practice and underline the importance of addressing the priorities and experiences of patients and families affected by familial dementia. 

## Supplementary Information

Supplementary Material 1.

Supplementary Material 2.

## Data Availability

The datasets used and/or analyzed during the current study are available from the corresponding author on reasonable request.

## Abbreviations

AD: Alzheimer’s disease
ALS: Amyotrophic lateral sclerosis
BBQ: Brunnsviken Brief Quality of Life Scale
COREQ: Consolidated Criteria for Reporting Qualitative Research
CSF: Optional cerebrospinal fluid
DLB: Dementia with Lewy bodies
DRS: Decision Regret Scale
FTD: Frontotemporal dementia
HADS: Hospital Anxiety and Depression Scale
HD: Huntington’s disease
IES: Impact of Event Scale
MCLHB-DRR: Motivation to Change Lifestyle and Health Behaviors for Dementia Risk Reduction Scale
MSPSS: Multidimensional Scale of Perceived Social Support
MRI: Magnetic resonance imaging
ODCF: Openness to Discuss Cancer in the Family
VaD: Vascular dementia
VAS: Visual analogue scale
VUS: Variant of uncertain significance
