# Supplementary material for: Impact of diagnostic genetic testing for familial dementia: experiences of patients and relatives
Source: Alzheimers Res Ther. 2026 Mar 17;18:92. doi: 10.1186/s13195-026-02000-z (PMC13107723; doi:10.1186/s13195-026-02000-z)
Supplement: Supplementary file 1 — Supplementary Material 1. [file 13195_2026_2000_MOESM1_ESM.docx]

**Supplement 1**

Interview guide topic list

## *Opening question*

How did you get involved with DNA testing?

### *Introduction*

- How were you told that you were eligible for DNA testing?
- What was your initial reaction when that option was presented to you?
- Why (for what purpose) was DNA testing offered to you?
- What question could DNA testing answer for you?

### *Decision-making*

- How did you reach your decision?
- To what extent did you discuss this with your family?
- How did they view the choice and consequences?
- How did you weigh your own interests with those of others?

### *Considerations*

- Why did you choose to (not) undergo DNA testing?
- What is the main purpose/added value of DNA testing for you?
- What considerations played a role in your decision?
  - Why do you think that is important?
- What do you see as the main benefits for yourself or your family?
  - Are there any other positive aspects?
- What do you see as main disadvantages for yourself or your family?
  - Are there any other negative aspects?
- Have considerations regarding treatment played any role in this?

*Test result*

- How does the test result affect you?
  - How did you feel upon receiving the test result?
  - What emotions have you experienced since?
- What does it mean for you and your family that a/no genetic cause is found?
- What do you see as the main consequences?
  - Only positive, or also negative?
- What else do you think about regarding the test result?

### *Stigma*

- Did you share DNA testing with others, outside your immediate family?
- How did they react?
- Do you think a genetic predisposition affects the way you view yourself?
- Do you think it affects how others perceive you?
- Do you think it affects the perception others have of your family?

### *Discrimination*

- To what extent do you think genetic testing has an influence on personal, professional, financial or legal aspects?
- (For example, relationships, work, insurance)

### *Actionability*

- Have you done or not done certain things based on the results?
- (For example, participation in research, nutrition (supplements), physical activity, cognitive activity, non-resuscitation / will / testament, changing plans, adjusting priorities, increasing quality of life)

### *Norms*

- Do you think people who are important to you would agree with your choice?
- What do you think others would do in your situation?
- Can you imagine a situation in which you would make a different choice?
- There are also people who make other choices. What kind of people would do or not do it right do you think?

### *Informed decision-making*

- How do you feel about this option of DNA testing being presented to you?
- How do you feel about being allowed to decide for yourself?
- How did you feel about the information and guidance you received about this?
- Did you have enough time and information to decide?
- To what extent do you feel you were able to make a voluntary, considered, and informed decision about it?

### *Other*

- Do you have anything else to add?
- Is there anything I haven't asked you that you think is relevant?
