# Supplementary material for: Impact of diagnostic genetic testing for familial dementia: experiences of patients and relatives
Source: Alzheimers Res Ther. 2026 Mar 17;18:92. doi: 10.1186/s13195-026-02000-z (PMC13107723; doi:10.1186/s13195-026-02000-z)
Supplement: Supplementary file 2 — Supplementary Material 2. [file 13195_2026_2000_MOESM2_ESM.docx]

**Supplement 2**

- *Actionability* was assessed using a self-developed six-item scale; participants rated agreement with each statement on a 5-point Likert scale (1=strongly disagree, 5=strongly agree).
  - I sought support in some way, for example through professional help, friends, faith, a hobby, a pet, relaxation, or something similar.
  - I took steps to prepare for the future (for example, arranging care, an advance directive, or a will).
  - I brought plans forward and adjusted priorities, to do now what matters to me because it may not be possible later.
  - I changed my lifestyle.
  - I signed up to participate in scientific research.
- *Sharing with and support from partner, family and others* was assessed using a self-developed six-item scale; participants rated agreement with each statement on a 5-point Likert scale (1=strongly disagree, 5=strongly agree).
  - I discussed the [possibility / result]* of DNA testing with my partner.
  - I found support from my partner in coping with this.
  - I discussed the [possibility / result]* of DNA testing with my family.
  - I found support from my family in coping with this.
  - I discussed the [possibility / result]* of DNA testing with others.
  - I found support from others in coping with this.

* [possibility] was used for the post-counseling assessment; [result] was used for the post-disclosure assessment.

These scales were self-constructed, drawing from literature on factors potentially associated with impact of DNA testing for genetically inherited disorders [1-15].

**References**

[1] Chen G, Sexton A. Genetic Counseling and Testing for Dementia – A Scoping Review of Patient and Relatives Experiences and Outcomes. Patient Education and Counseling. 2025:109424.

[2] Crook A, Jacobs C, Newton-John T, Richardson E, McEwen A. Patient and Relative Experiences and Decision-making About Genetic Testing and Counseling for Familial ALS and FTD: A Systematic Scoping Review. Alzheimer Dis Assoc Disord. 2021.

[3] Rolf B, Blue EE, Bucks S, Dorschner MO, Jayadev S. Genetic counseling for early onset and familial dementia: Patient perspectives on exome sequencing. J Genet Couns. 2021;30:793–802.

[4] Rutherford HA, Rush BK, Smith A, Sullivan E, Martinez-Rubio C, Toumadj A, et al. Mapping the journey of patients and care partners living with adult-onset leukoencephalopathy with axonal spheroids and pigmented glia: developing a framework for improvements in care. Neurodegenerative Disease Management. 2024;14:161–72.

[5] Akrich M, Paterson F, Rabeharisoa V. Living with the rare late-onset genetic disease CADASIL: Improvising “tactics” to appropriate biomedical knowledge and technology. Social Science & Medicine. 2025;369:117797.

[6] Hess PG, Preloran HM, Browner CH. Diagnostic genetic testing for a fatal illness: the experience of patients with movement disorders. New Genetics and Society. 2009;28:3–18.

[7] Lewit-Mendes MF, Lowe GC, Lewis S, Corben LA, Delatycki MB. Young People Living at Risk of Huntington’s Disease: The Lived Experience. Journal of Huntington's Disease. 2018;7:391–402.

[8] Wagner KN, Nagaraja HN, Allain DC, Quick A, Kolb SJ, Roggenbuck J. Patients with sporadic and familial amyotrophic lateral sclerosis found value in genetic testing. Mol Genet Genomic Med. 2018;6:224–9.

[9] Huq AJ, Sexton A, Lacaze P, Masters CL, Storey E, Velakoulis D, et al. Genetic testing in dementia-A medical genetics perspective. Int J Geriatr Psychiatry. 2021;36:1158–70.

[10] Bleiker EM, Esplen MJ, Meiser B, Petersen HV, Patenaude AF. 100 years Lynch syndrome: what have we learned about psychosocial issues? Fam Cancer. 2013;12:325–39.

[11] Coustasse A, Pekar A, Sikula A, Lurie S. Ethical considerations of genetic presymptomatic testing for Huntington's disease. J Hosp Mark Public Relations. 2009;19:129–41.

[12] Crook A, Jacobs C, Newton-John T, McEwen A. Genetic counseling and diagnostic genetic testing for familial amyotrophic lateral sclerosis and/or frontotemporal dementia: A qualitative study of client experiences. J Genet Couns. 2022;31:1206–18.

[13] Høberg-Vetti H, Eide GE, Siglen E, Listøl W, Haavind MT, Hoogerbrugge N, et al. Cancer-related distress in unselected women with newly diagnosed breast or ovarian cancer undergoing BRCA1/2 testing without pretest genetic counseling. Acta Oncologica. 2019;58:175–81.

[14] Licklederer C, Wolff G, Barth J. Mental health and quality of life after genetic testing for Huntington disease: a long-term effect study in Germany. Am J Med Genet A. 2008;146a:2078–85.

[15] Largent EA, Harkins K, van Dyck CH, Hachey S, Sankar P, Karlawish J. Cognitively unimpaired adults' reactions to disclosure of amyloid PET scan results. PLoS One. 2020;15:e0229137.
